# Urea Coated with Polyaspartic Acid-Chitosan Increases Foxtail Millet (*Setaria italica* L. Beauv.) Grain Yield by Improving Nitrogen Metabolism

**DOI:** 10.3390/plants13030415

**Published:** 2024-01-31

**Authors:** Lin Lu, Qi Wang, Wei Zhang, Ming Gao, Yanli Xv, Shujie Li, Haosheng Dong, Disu Chen, Peng Yan, Zhiqiang Dong

**Affiliations:** 1 Institute of Crop Sciences, Chinese Academy of Agricultural Sciences, Beijing 100081, China; lulin@caas.cn (L.L.); veraz0208@163.com (W.Z.); xuyanli412@163.com (Y.X.); 18394792517@139.com (H.D.); 18309318033@163.com (D.C.); 2 Beijing Agricultural Technology Extension, Beijing 100029, China; wangqi240@126.com; 3 College of Resources and Environment, Shanxi Agricultural University, Jinzhong 030801, China; 4 Institute of Crop Resources Sciences, Jilin Academy of Agricultural Sciences, Changchun 130033, China; gaomingchina@163.com (M.G.); shujie-li@163.com (S.L.)

**Keywords:** fertilizer, nitrate reductase, nitrogen availability, high yield and efficiency, one-time basic fertilizer application

## Abstract

Innovative measures of nitrogen (N) fertilization to increase season-long N availability is essential for gaining the optimal foxtail millet (*Setaria italica* L. Beauv.) productivity and N use efficiency. A split plot field experiment was conducted using the foxtail millet variety Huayougu 9 in 2020 and 2021 in Northeast China to clarify the physiological mechanism of a novel polyaspartic acid–chitosan (PAC)-coated urea on N assimilation and utilization from foxtail millet. Conventional N fertilizer (CN) and the urea-coated -PAC treatments were tested under six nitrogen fertilizer application levels of 0, 75, 112.5, 150, 225, and 337.5 kg N ha^−1^. The results showed that compared to CN, PN increased the foxtail millet yield by 5.53–15.75% and 10.43–16.17% in 2020 and 2021, respectively. PN increased the leaf area index and dry matter accumulation by 7.81–18.15% and 12.91–41.92%, respectively. PN also enhanced the activities of nitrate reductase, glutamine synthetase, glutamic oxaloacetic transaminase, and glutamic–pyruvic transaminase, thereby increasing the soluble protein in the leaf, plant, and grain N content at harvest compared to CN. Consequently, partial factor productivity from applied N, the agronomic efficiency of applied N, recovery efficiency of applied N, and physiological efficiency of applied N of foxtail millet under PN treatments compared to CN were increased. The improvement effect of the items above was more noticeable under the low–middle N application levels (75, 112.5, and 150 kg N ha^−1^). In conclusion, the PAC could achieve the goal of high yield and high N use efficiency in foxtail millet under the background of a one-time basic fertilizer application.

## 1. Introduction

Foxtail millet (*Setaria italica* L. Beauv.) is an ancient crop in the subfamily of *Poaceae*, and is mainly cultivated in semi-arid regions of India, China and other parts of Asia, North Africa, and America. Foxtail millet originated in North China, and was domesticated more than 11,000 years ago [1]; it was praised as “the feeding crop of Chinese civilization”. Foxtail millet has a number of characteristics in relation to its physiological and ecological aspects such as a strong tolerance to drought and to poor soil, including levels of saline–alkali. Based on the above advantages, foxtail millet has become the main crop in Chinese agriculture [2]. Nitrogen is an essential and limiting nutrient factor for foxtail millet yield and quality. Foxtail millet is mainly distributed in the semi-arid regions and barren areas in China, where the nitrogen fertilizer use efficiency is quite low. Additionally, due to the shortage of labor force in rural areas, Chinese farmers generally apply a large amount of base fertilizer at one time and no topdressing at a later stage. Although this method can save time and labor, it causes the uneven distribution of nitrogen in the whole growth period of crops; that is, distribution is excessive in the early stages and insufficient in the later stages, which is not in line with the crop’s fertilizer demand [3]. Therefore, it is of great practical significance to explore how to improve the nitrogen use efficiency under the background of a one-time basic fertilizer application to achieve the target of high yield and efficiency.

Higher plants cannot use nitrogen from the air directly, but can only absorb combined nitrogen such as ammonium and nitrate. Nitrate is the main nitrogen source for dryland crops. NO_3_^−^ is first reduced to nitrite by nitrate reductase (NR) and then further to ammonium by nitrite reductase (NiR) and glutamine synthetase (GS). The ammonium derived from nitrate or directly from ammonium uptake by ammonium transporters (AMTs) is further assimilated into amino acids via the GS/glutamine-2-oxoglutarate aminotransferase (GOGAT) cycle. The glutamate (Glu) amino group can be transferred to other kinds of amino acids by a number of different aminotransferases such as glutamic oxaloacetic transaminase (GOT), glutamic-pyruvic transaminase (GPT), and so on [4]. As the final product of nitrogen metabolism, soluble protein content can be used as a reflection of nitrogen metabolism ability. Good nitrogen metabolism and nitrogen use in the middle and late growth stages of crops could effectively delay the process of plant senescence, which is conducive to maintaining a high leaf area index and promoting the accumulation of photosynthates. Flag leaves are the part where the metabolism is the most active in crops in the late growth period, which makes an important contribution to yield formation [5]. It is well demonstrated that enhancing the activities of enzymes in nitrogen metabolism and increasing their product content in flag leaves after anthesis usually directly results in higher nitrogen accumulation, nitrogen utilization efficiency, and final yield.

Fertilizer synergists are active substances which could increase the nutrient availability and have been widely used in agricultural production in recent years. Polyaspartic acid–chitosan (PAC) is a new fertilizer synergist which is composed of polyaspartic acid (PASP) and chitosan (CTS). PASP is an ecofriendly and nontoxic polyamine acid that possesses carboxylic groups with good chelating ability and dispersibility. Also, PASP has notable properties of low production costs and biodegradation [6]. Because of the advantages above, PASP has been widely applied in agriculture as the absorption promoter of fertilizers, a controlled-release agent, and so on [7,8]. CTS is a derivative of chitin and is considered the second most common polymer in the world after cellulose [9]. CTS has generated great attention in a range of fields due to its exceptional biological activities, such as biodegradability [10], biocompatibility [11], non-toxicity [12], and antimicrobial activity [13]. In agriculture, CTS is used through foliar application on plants [14], as a seed treatment [15], or as a direct soil fertilizer [16]. A significant number of previous studies aiming to explain the effects of PASP and CTS on crop growth and development have mostly focused on bulk grain crops and cash crops, and they were conducted independently. However, information about the effects of PAC (combined PASP and CTS) on foxtail millet growth and nitrogen assimilation, especially its physiological mechanism, remains limited, in particular when combined with the one-time basal application method.

Thus, the aim of this study was to explore the regulatory effects of PAC on the nitrogen metabolic enzyme activities of flag leaf, nitrogen utilization, and yield of foxtail millet, and to clarify the physiological mechanism of PAC-regulating nitrogen absorption, assimilation, and utilization, so as to provide a theoretical and technical basis for the high yield and efficiency of foxtail millet under the background of the one-time basic fertilizer application.

## 2. Results

### 2.1. Grain Yield and Yield Components

The year, PAC, N level, and the interactions (PAC × N level, PN) demonstrated significant effects on grains per ear and yield of foxtail millet. As shown in Table 1, N rates increased the yield significantly. In comparison with N0, the yield from N75 to N337.5 increased by 65.68–104.35% in 2020 and 30.68–73.18% in 2021, respectively. PN treatment increased the yield by 5.53–15.75% and 10.43–16.17% in comparison with CN in 2020 and 2021, respectively, with nitrogen application rates from N75 to N337.5. The highest grain yields were both obtained from the N150 with PAC treatment in two years.

Analysis of yield components showed that PN treatments significantly increased the number of grains per ear of foxtail millet by 9.26–61.20% and 18.83–20.62% compared with CN in 2020 and 2021, respectively, with nitrogen application rates from N75 to N337.5. However, no significant difference was observed between CN and PN treatments in relation to the 1000-grain weight and ears per unit area.

### 2.2. Leaf Area Index and Dry Matter Accumulation

**Figure 1:**
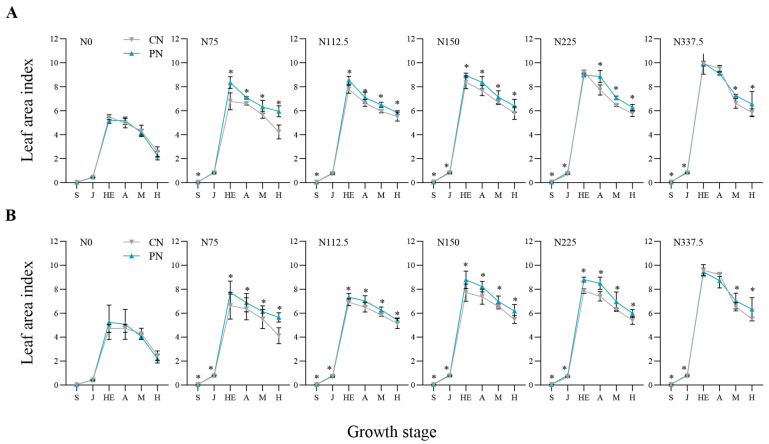
Effects of CN and PN treatments on leaf area index of foxtail millet under different nitrogen application levels in 2020 (**A**) and 2021 (**B**). CN: conventional nitrogen fertilizer treatment; PN: polyaspartic acid–chitosan with nitrogen fertilizer treatment. N0, N75, N112.5, N150, N225, and N337.5 represent nitrogen application amounts of 0, 75, 112.5, 150, 225, and 337.5 kg N ha^−1^, respectively. S: seeding stage; J: jointing stage; HE: heading stage; A: anthesis stage; M: mid-filling stage; H: harvesting stage. * represents significant difference at *p* < 0.05 according to *t*-test.

**Figure 2:**
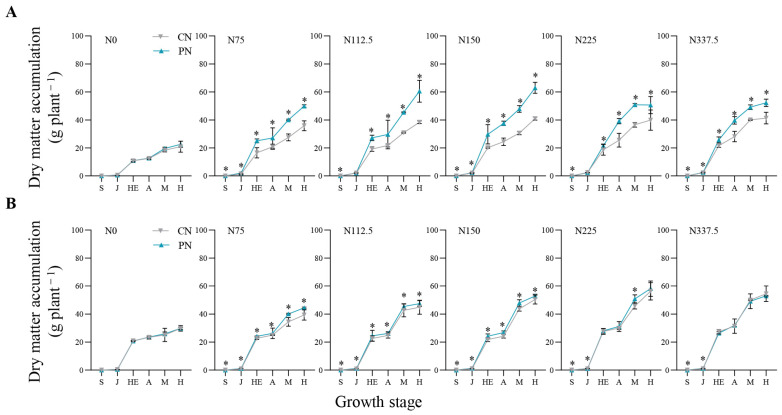
Effects of CN and PN treatments on dry matter accumulation of foxtail millet under different nitrogen application levels in 2020 (**A**) and 2021 (**B**). CN: conventional nitrogen fertilizer treatment; PN: polyaspartic acid–chitosan with nitrogen fertilizer treatment. N0, N75, N112.5, N150, N225, and N337.5 represent nitrogen application amount of 0, 75, 112.5, 150, 225, and 337.5 kg N ha^−1^, respectively. S: seeding stage; J: jointing stage; HE: heading stage; A: anthesis stage; M: mid-filling stage; H: harvesting stage. * represents significant difference at *p* < 0.05 according to *t*-test.

N rates and PN both showed significant effects on the leaf area index (LAI) (Figure 1) and dry matter accumulation (DMA) (Figure 2) of foxtail millet in the two years. Compared with N0, the DMA and LAI under N75–N337.5 increased by 49.53–329.16% and 24.11–154.64% in 2020, and by 3.49–174.29% and 28.94–157.67% in 2021, respectively. The effect of PN on LAI and DMA depended more on the foxtail millet growth stages and N rates. In 2020, compared with CN, PN increased LAI significantly from seedling to harvesting stages, leading to 18.15%, 7.81%, 9.56%, and 14.06% increases on average from seedling to harvesting stages under N75 to N225, except the ones at jointing stage under N75 and N112.5 and at heading stage under N225 (Figure 1A). Meanwhile, in 2021, PN on average increased LAI by 14.30%, 6.73%, 11.76%, 14.10%, and 11.30% from seedling to harvesting stages significantly under N75 to N337.5, except the ones at heading and anthesis stages under N337.5 (Figure 1B).

In 2020, compared with CN, PN increased DMA at all sampled growth stages on average by 38.30%, 39.88% 41.92%, 30.58%, and 21.59% from N75 to N337.5, except the ones at jointing stage under N112.5 and N225 (Figure 2A). And in 2021, there were 15.78%, 12.91%, and 14.99% increases on average on DMA under N75, N112.5 and N150; PN averagely increased DMA by 14.73% at seedling, jointing and mid-filling stages under N225; PN increased DMA by 11.81% and 14.70% at seedling and jointing stages under N337.5, respectively (Figure 2B).

### 2.3. Activities of NR, GS, GOT, and GPT

**Figure 3:**
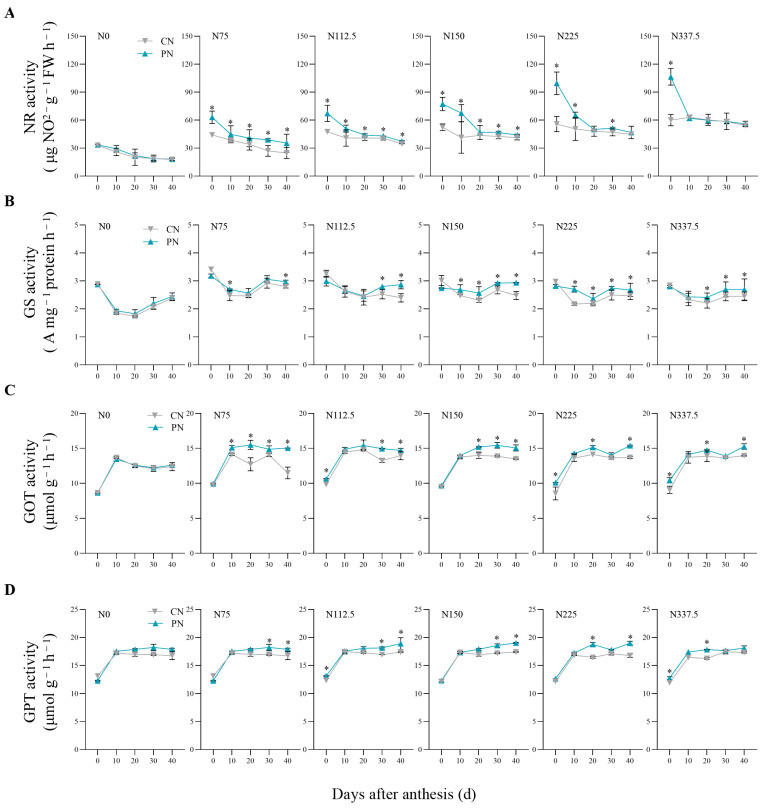
Effects of CN and PN treatments on key enzymatic activity of nitrogen assimilation of foxtail millet leaf under different nitrogen application levels. CN: conventional nitrogen fertilizer treatment; PN: polyaspartic acid–chitosan with nitrogen fertilizer treatment. N0, N75, N112.5, N150, N225, and N337.5 represent nitrogen application amount of 0, 75, 112.5, 150, 225, and 337.5 kg N ha^−1^, respectively. * represents significant difference at *p* < 0.05 according to *t*-test. (**A**) NR activity. (**B**) GS activity. (**C**) GOT activity. (**D**) GPT activity.

N rates and PN both showed significant effects on NR activity (Figure 3A) and GS activity (Figure 3B) of flag leaf of foxtail millet in the 0–40 days after anthesis. Under N75, N112.5, and N150, compared with CN, PN on average significantly increased NR activity by 32.98%, 17.62%, and 26.94% in the 0–40 days after anthesis, respectively; PN treatment on average significantly increased NR activity by 39.03% at 0, 10, and 30 days after anthesis under N225 and by 77.44% in anthesis (0 day) under N337.5 (Figure 3A). Compared with CN, PN treatments on average significantly increased GS activity by 11.84% and 12.47% at 10–40 days after anthesis under N150 and N225, respectively (Figure 3B).

During the 0–40 days after anthesis, GPT activity (Figure 3C) and GOT activity (Figure 3D) of flag leaves of foxtail millet differed significantly among CN and PN, while the N level had no significant effect on them. Compared with CN, PN treatments significantly averagely increased GOT activity by 16.35% in the 10–40 days after anthesis under N75, by 8.52% at 0, 30, and 40 days after anthesis under N112.5, and by 10.41% at 20–40 days after anthesis under N150; PN treatments had 12.56% and 10.17% significantly higher values for GOT activity on average at 0, 20, and 40 days after anthesis under N225 and N337.5, respectively (Figure 3C). Compared with CN, PN on average significantly increased GPT activity by 7.89% and 7.92% at 30 and 40 days after anthesis under N75 and N150; PN on average significantly increased GPT activity by 7.49% at 0, 30, and 40 days after anthesis under N112.5; there were 14.20% and 13.23% increases in GPT activity at 20 and 40 days after anthesis under N225, and 7.04% and 9.65% increases at 0 and 20 days after anthesis under N337.5 (Figure 3D).

### 2.4. Soluble Protein Content

**Figure 4:**
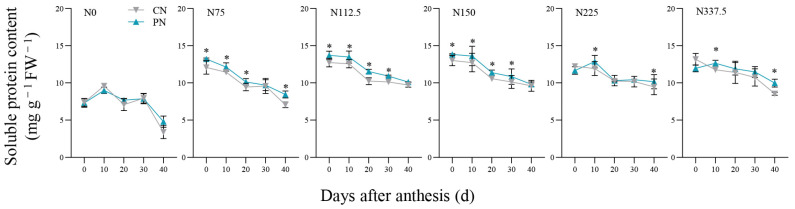
Effects of CN and PN treatments on soluble protein content of foxtail millet leaf under different nitrogen application levels. CN: conventional nitrogen fertilizer treatment; PN: polyaspartic acid–chitosan with nitrogen fertilizer treatment. N0, N75, N112.5, N150, N225, and N337.5 represent nitrogen application amount of 0, 75, 112.5, 150, 225, and 337.5 kg N ha^−1^, respectively. * represents significant difference at *p* < 0.05 according to *t* test.

Nitrogen rates and PN both showed significant effects on the soluble protein content of the flag leaves of foxtail millet during the 0–40 days after anthesis (Figure 4). Compared with CN, PN significantly increased the soluble protein content by 10.58% at 0, 10, 20, and 40 days after anthesis under N75; the soluble protein contents during the 0–30 days after anthesis under N112.5 and N150 were significantly increased by 8.77% and 7.07%, respectively; the soluble protein content at 10 and 40 days after anthesis under N225 and N337.5 were significantly increased by 7.55% and 12.17%, respectively.

### 2.5. Total Nitrogen Accumulation in the Plant and Grain

**Figure 5:**
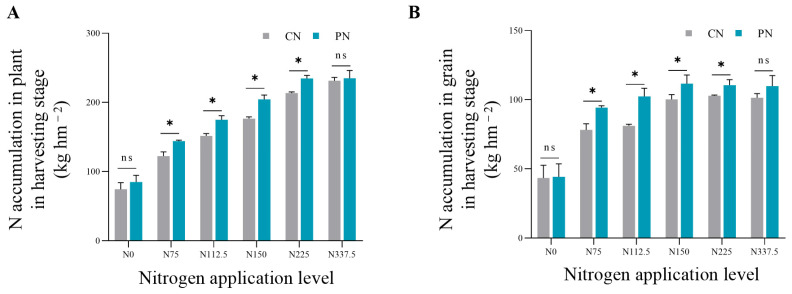
Effects of CN and PN treatments on total nitrogen accumulation in plant and grain of foxtail millet under different nitrogen application levels. (**A**,**B**) represent total nitrogen accumulation in plant and grain, respectively. CN: conventional nitrogen fertilizer treatment; PN: polyaspartic acid–chitosan with nitrogen fertilizer treatment. N0, N75, N112.5, N150, N225, and N337.5 represent nitrogen application amount of 0, 75, 112.5, 150, 225, and 337.5 kg N ha^−1^, respectively. * represents significant difference at *p* < 0.05, and ns represents no significant difference.

As shown in Figure 5, compared with N0, the total nitrogen accumulation in the plant and grain at harvest increased by 64.56–215.83% and 79.83–156.97%, respectively, under N75–N337.5. Compared with CN, PN treatments on average significantly increased the total N accumulation in plant at harvesting stage by 14.63% under N75–N225; and PN on average significantly increased the N accumulation in grain by 19.43% under N75, N112.5, and N150.

### 2.6. Nitrogen Use Efficiency

At harvest, as shown in Table 2, the N application rates and PN had significant effects on nitrogen use efficiency of foxtail millet. NPFP, NAE, and NPE tended to decrease as the nitrogen fertilizer rate increased, while NRE tended to increase at first and then decrease. Compared with CN, PN treatments significantly increased NPFP and NAE by 11.17% and 35.04% on average in 2020 under N75, N112.5, and N150 kg, respectively, and by 12.85% and 42.63% on average in 2021 under N75 to N337.5, respectively. PN significantly increased NRE by 15.83% and 30.14% on average under N75–N337.5 in 2020 and 2021, respectively, except the one in 2020 under N337.5. Also, PN significantly enhanced NPE, resulting in a 19.03% improvement on average under N112.5–N337.5 in 2020, and 8.17% and 9.31% improvements under N75 and N112.5 in 2021, respectively.

## 3. Discussion

Nitrogen is one of the essential elements of crops, which contributes 40–50% to yield [17]. The grain yield tends to increase with the fertilizer rate within limits, while at high fertilizer levels, excessive vegetative growth and serious lodging may be responsible for yield loss [18,19]. The results in this study indicated that the yield of foxtail millet increased first and then decreased with the increase in nitrogen application rate (Table 1), which is in agreement with the results of previous studies [20]. Both PASP and CTS could promote crop growth and increase yield effectively [15,21]. Data in our study showed that compared with CN, PN increased the yield of foxtail millet by 5.53–15.75% and 10.43–16.17% in 2020 and 2021, respectively (Table 1). Ears per unit area, grains per ear, and 1000-grain weight are three components of yield of foxtail millet. This study found that PN had no significant effects on 1000-grain weight and ears per unit area (Table 1). The 1000-grain weight of foxtail millet was different in the two years, probably because of the attack of foxtail millet blast and downy, mildew, the 1000-grain weight decreased in 2021. But PAC played a certain role in promoting the increase in 1000-grain weight under partial nitrogen application compared with CN, which indicated that PAC could enhance the ability of foxtail millet to resist diseases. Thus, the adverse effect of pathogens on grain filling was overcome and a high and stable yield was guaranteed. This may be credited to the antibacterial ability of CTS, one of the main components of PAC. However, the specific mechanism remains to be further explored. Our study showed that PAC increased grains per ear significantly under the low–middle nitrogen application levels (N75, N112.5, and N150) (Table 1), indicating that it could increase the yield of foxtail millet by increasing the grains per ear under the appropriate nitrogen application rate, so as to realize the goal of tapping high yield potential.

Leaf area index (LAI) could reflect the canopy structure and light energy utilization of crops. Higher LAI is conducive to the formation and accumulation of photosynthates [22]. We found that PN could significantly increase the LAI of foxtail millet (Figure 1), thus effectively promoting photosynthesis. Dry matter is the final product of photosynthesis, which is positively correlated with yield. The formation of grain weight is mainly determined by the accumulation and transfer of dry matter [23]. The results of this experiment showed that PN could significantly increase the DMA of foxtail millet under the low–middle nitrogen application levels (N75, N112.5, and N150) (Figure 2); thus, laying the foundation for the improvement of economic yield.

Nitrate reductase (NR) is the first rate-limiting enzyme that regulates nitrogen assimilation and transport in the leaf of most crops [24]. In this study, as the nitrogen application rate was increased, the NR activity of flag leaves of foxtail millet first increased; however, when the nitrogen application rate was increased to a certain level, NR activity began to decrease (Figure 3). It indicated that excessive nitrogen was unfavorable for the improvement of nitrogen metabolic enzyme activities, which is consistent with the research conclusions of Ma et al. [25] on maize. Compared with CN, PN increased NR activity of foxtail millet under the low–middle nitrogen application levels (N75, N112.5, and N150) (Figure 3A). Previous research has made it clear that NR activity was positively correlated with the concentration of NO_3_^−^, the substrate of the reaction [26]. So, PN promoted nitrogen uptake and provided essential conditions for plant growth and efficient nitrogen utilization. Other studies have shown that higher NR activity after anthesis could regulate leaf senescence [27]. Therefore, PAC could prolong the functional period of leaves, so as to promote the accumulation of dry matter in post-anthesis and yield formation.

Glutamine synthetase (GS) is a key synthetase located in the center of nitrogen metabolism, which can assimilate NH_4_^+^ absorbed from the outside and produced through various internal nitrogen cycling pathways, and reduce the toxic effect of ammonium [28]. The increase in GS activity at a late growth stage can promote nitrogen reactivation, which is a direct reflection of enhanced nitrogen metabolism [25]. The results of this study showed that compared with CN, PAC could significantly enhance GS activity at the middle and late stages of grain filling under the same nitrogen application level (Figure 3B), meaning that PAC could accelerate the nitrogen metabolism of foxtail millet at the late growth stage.

Glutamic oxaloacetic transaminase (GOT) and glutamic-pyruvic transaminase (GPT) could regulate nitrogen transfer from glutamic acid to other carbon chains and play a role in amino acid reuse at a late growth stage [29]. Our results showed that the activities of GOT and GPT increased first and then remained unchanged as time went by, which might be related to the requirements of aspartic acid and alanine in foxtail millet in post-anthesis. PAC could significantly increase the activities of GOT and GPT at the middle and late stages of grain filling, so as to provide sufficient amino acid donors for protein synthesis (Figure 3C,D). Soluble protein is closely related to nitrogen metabolism and senescence of plants [30]. The results of this experiment showed that the soluble protein content after anthesis increased first and then decreased with the increase in the nitrogen application rate, which was consistent with the findings reported by Lu et al. [31]. The increase effect was also more marked under the low–middle nitrogen application levels (N75, N112.5, and N150) (Figure 4).

Increased activity of nitrogen metabolizing enzymes can directly promote nitrogen absorption and assimilation, thus enhancing nitrogen accumulation. Studies have shown that grain yield mainly depends on nutrient accumulation and nutrient transport from vegetative organs in post-anthesis [32], which depends on pre-anthesis nutrient accumulation. PAC increased nitrogen accumulation of foxtail millet under the low-middle nitrogen application levels of N75, N112.5, and N150 significantly at harvesting stage (Figure 5A), indicating that PAC had a more obvious sustained-release effect under low nitrogen condition, which could enhance the nitrogen supply in post-anthesis and significantly improve the nitrogen accumulation of plants, thus effectively solving the problem of insufficient nitrogen supply during the late growth period of foxtail millet under the background of a one-time basic fertilizer application. Increasing grain protein content is a vital way of improving grain nutrient quality. Nitrogen is an important component of protein. Therefore, higher nitrogen accumulation in grain is a guarantee of the high quality of crops [33]. This study showed that PAC combined with conventional nitrogen fertilizer could increase the nitrogen accumulation in grain (Figure 5B), which indicated that PAC was beneficial to the transport of nitrogen to grains, thus resulting in better grain quality.

The realization of the effect of nitrogen fertilizer on yield increase mainly depends on the utilization rate of fertilizer in the current season; therefore, improving nitrogen use efficiency has always been the main goal of crop improvement [34]. Higher nitrogen use efficiency can promote crop growth and accumulation of photosynthetic products, and ultimately increase yield under the same nitrogen application rate. Our results showed that PN could significantly enhance NPFP, NAE, NRE, and NPE (Table 2), indicating that PN can improve the nitrogen utilization potential of foxtail millet and reduce nitrogen loss, so as to achieve both economic and ecological benefits.

PASP, one of the components of PAC, contains a large number of active groups such as the amino group, carboxyl group, and hydroxyl group, which has good chelation, adsorption, and sustained-release effects. It could chelate a variety of nutrient elements, promote the absorption and utilization of fertilizer, and effectively promote the nitrogen metabolism of crops. Recent research has indicated that PASP combined with urea could increase nitrogen accumulation in the upper part of sorghum, and improve nitrogen use efficiency and grain yield [8]; increase NR activity and promote nitrogen assimilation in maize seedlings [35]; and increase the activities of enzymes of nitrogen metabolism and the content of soluble protein in functional leaves of rice [21]. CTS, the other component of PAC, also has good adsorption and sustained-release effects, which can accelerate life activities of microorganisms in soil, promote soil respiration, improve nutrient availability [9], and the fertilizer absorption efficiency of plants [16]. Also, CTS can increase grain nitrogen accumulation and improve grain quality [36]. Studies have suggested that CTS could effectively improve the activities of nitrogen metabolic enzymes of leaves in wheat [37] and okra [38]. Therefore, PAC combined with nitrogen fertilizer application could increase the activities of key enzymes and the content of nitrogen metabolism products of leaf of foxtail millet in post-anthesis, promote nitrogen metabolism during the grain filling period, thus improving crop nitrogen uptake, fertilizer absorption, and utilization efficiency, promoting photosynthate accumulation, and finally increasing yield.

## 4. Materials and Methods

### 4.1. Plant Materials and Experimental Design

**Figure 6:**
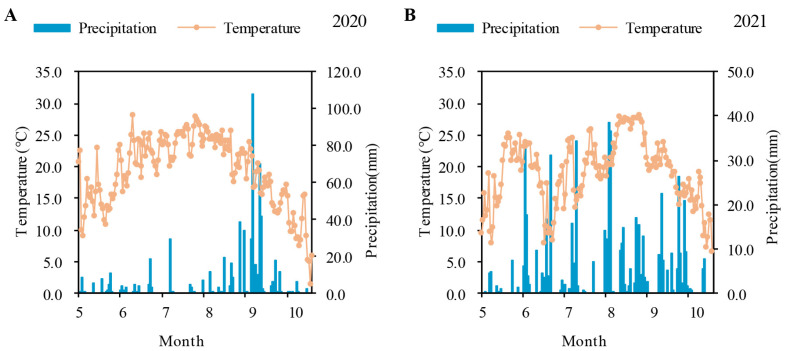
Daily precipitation and mean temperatures during growing seasons of foxtail millet in 2020 (**A**) and 2021 (**B**).

The field experiments were conducted at the Gongzhuling Experimental Station of Chinese Academy of Agricultural Sciences (43°29′55″ N, 124°48′43″ E) in Jilin, China. The soil was black soil with 26.7 g kg^−1^ organic matter, 1.4 g kg^−1^ total nitrogen, 155.3 mg kg^−1^ available nitrogen, 34.4 mg kg^−1^ available phosphorus, and 184.2 mg kg^−1^ available potassium. The soil pH was 5.8. The daily mean temperature and precipitation during the rice growing season (May to October) are shown in Figure 6. Foxtail cultivar Huayougu 9, bred by the Institute of Crop Sciences, Chinese Academy of Agricultural Sciences, was used in this study.

Plots were distributed in a randomized complete block design with three replicates in 2020 and 2021. Treatments included two urea treatments, a control treatment (CN, uncoated urea), and polyaspartic acid–chitosan-coated urea (PN). Both urea treatments consisted of six N rates: 00, 75, 112.5, 150, 225, and 337.5 kg N ha^−1^. The method of fertilizer application was a one-time base application. PN was made in the laboratory: first, a prepared poly (aspartic acid) (PAA) solution was mixed with poly (succinimide) powder (obtained by acid-catalyzed polycondensation of L-aspartic acid) with distilled water, and KOH was added until the poly (succinimide) was completely dissolved. The glacial acetic acid was added to distilled water, and glacial acetic acid solution was added until the chitosan (CTS) was completely dissolved. Second, urea was coated with PAA solution at 0.3% of the total urea rate and then air-dried naturally in the shade, followed by being coated with CTS solution at 0.45% of the total urea rate and continuing to be air-dried naturally in the shade.

The dimension of each plot was 6.0 × 4.2 m. The seedling density of foxtail millet was 600,000 ha^−1^, and the row spacing was 0.6 m. In all treatments, 75 kg ha^−1^ of P_2_O_5_ and 180 kg ha^−1^ of K_2_O were applied at the basal stage. Field management measures such as intertillage, weeding, and plant protection were the same as local field production. Foxtail millet was sown on 6 May and harvested on 17 September.

### 4.2. Sampling and Measurement

#### 4.2.1. Yield and Yield Components

At harvest, 3 m^2^ in the inner four rows of each plot was harvested to measure grain yield. Plant density was measured as the number of plants per square meter. Grain moisture content was determined using a PM-8188 grain moisture analyzer (Tokyo, Japan) and measured 10 times for each sample. Grain from the sampling area was weighed and corrected to a water content of 12% to calculate the final sorghum grain yield.

#### 4.2.2. Leaf Area Index and Dry Matter Accumulation

The leaf area of all leaves of five representative plants sampled from the middle of each plot at the same stages as dry matter production was measured, and the leaf area index (LAI) was calculated using the following formula: LAI = total leaf area per unit land area/land area. Three representative plants sampled from the middle of each plot were used to determine the dry matter accumulation (DMA) after oven-drying at 105 °C for 1 h and then at 85 °C until a constant weight at seeding stage, jointing stage, heading stage, anthesis stage, and harvesting stage.

#### 4.2.3. Determination of Enzyme Activities Related to Nitrogen Metabolism

Five flag leaves were sampled from each plot at 0, 10, 20, 30, and 40 days after anthesis. The midrib of the leaf was removed and its lamina was sliced and frozen in liquid N, and then kept at −80 °C until further analysis. NR and GS activities in the flag leaves were determined using the methods of Wang et al. [35]. GOT and GPT activities in the flag leaves were determined using the method of Wu et al. [39]. Soluble protein content was determined using the methods of Marion [40].

#### 4.2.4. Net Assimilation Rate

Total nitrogen accumulation (NA) in plants and grains was measured using elemental analyzer (Vario PYRO; Elementar, Germany). The methods described by Kenneth et al. [41] were used to calculate N partial factor productivity (NPFP), N agronomic efficiency (NAE), N recovery efficiency (NRE), and N physiological efficiency (NPE) as follows: NPFP (kg kg^−1^) = GY_N_/N fertilizer rate; NAE (kg kg^−1^) = (GY_N_ − GY_zero_)/N fertilizer rate; NRE (%) = (NA_N_ − NA_zero_)/N fertilizer rate × 100%; NPE (kg kg^−1^) = (GY_N_ − GY_zero_)/(NA_N_ − NA_zero_).

### 4.3. Data Analysis

In this study, 2-year data of yield and net assimilation rate were presented, and the average of 2-year data was used for other indicators. Microsoft Excel 2019 was used to organize, calculate, and chart the data. IBM SPSS Statistics 17.0 (IBM Inc., Chicago, IL, USA) was used for statistical analysis. Analysis of variance was performed with IBM SPSS Statistics 17.0 (IBM Inc., Chicago, IL, USA) to test the effects of year, treatments (CN and PN), and N rates on grain yield, agronomic traits, N content, and nitrogen use efficiency. The DUNCAN method and paired *t*-test (*p* < 0.05) were used to determine the differences among the treatments.

## 5. Conclusions

Compared with conventional nitrogen fertilizer, under the same nitrogen application rate, polyaspartic acid–chitosan (PAC) combined with conventional nitrogen fertilizer could increase the activities of nitrate reductase glutamine synthetase, glutamic oxaloacetic transaminase, glutamic-pyruvic transaminase, and the content of soluble protein of flag leaves of foxtail millet after anthesis under the low–middle nitrogen application levels (75, 112.5, and 150 kg N ha^−1^). In addition, PAC could significantly enhance the leaf area index and dry matter accumulation of foxtail millet, and improve the accumulation of the whole plant and grain; thus, strengthening the nitrogen supply at the later growth stage of foxtail millet and finally increasing the nitrogen fertilizer utilization efficiency and yield under the background of the one-time basic fertilizer application. The yield increased by 5.53–15.75% and 10.43–16.17% in 2020 and 2021, respectively. Based on the results above, PAC can achieve the high yield and efficiency of foxtail millet under the background of the one-time basic fertilizer application.

## Figures and Tables

**Table 1:** Effects of CN and PN treatments on yield and yield components of foxtail millet under different nitrogen application levels.

Year	Nitrogen Application Level	Treatment	1000-Grain Weight (g)	×10^4^ Ears ha^−1^	Grains per Ear	Yield (kg ha^−1^)
2020	N0	CN	2.8 ± 0.1 a	42.9 ± 2.8 c	3259.6 ± 294.1 f	2841.9 ± 266.1 e
		PN	2.9 ± 0.0 a	44.1 ± 1.4 c	3179.9 ± 397.0 f	2665.0 ± 576.4 e
	N75	CN	3.0 ± 0.0 a	48.4 ± 5.5 bc	3762.9 ± 276.6 ef	4819.2 ± 162.5 cd
		PN	2.8 ± 0.2 a	47.3 ± 1.4 bc	5627.1 ± 483.3 abc	5102.2 ± 41.8 bcd
	N112.5	CN	2.9 ± 0.1 a	48.9 ± 0.6 bc	3704.0 ± 110.1 ef	4708.3 ± 40.6 d
		PN	2.7 ± 0.2 a	54.2 ± 5.3 ab	5970.8 ± 488.0 ab	5449.6 ± 182.0 ab
	N150	CN	2.8 ± 0.0 a	54.3 ± 0.0 ab	4261.0 ± 488.5 def	5191.2 ± 319.8 bcd
		PN	2.9 ± 0.0 a	61.8 ± 0.0 a	4655.5 ± 346.7 cde	5807.4 ± 189.4 a
	N225	CN	2.9 ± 0.0 a	49.4 ± 0.4 bc	4304.0 ± 28.8 def	5322.4 ± 16.2 abc
		PN	2.7 ± 0.0 a	48.8 ± 1.1 bc	4989.9 ± 134.5 bcd	5573.2 ± 114.5 ab
	N337.5	CN	2.9 ± 0.1 a	44.4 ± 1.5 c	5634.9 ± 224.4 abc	5358.6 ± 90.6 abc
		PN	2.8 ± 0.1 a	47.3 ± 1.4 bc	6178.4 ± 152.8 a	5654.9 ± 225.7 ab
2021	N0	CN	2.8 ± 0.0 a	31.6 ± 3.4 e	2715.2 ± 69.1 f	3394.5 ± 321.8 e
		PN	2.8 ± 0.0 ab	33.3 ± 1.1 cde	2738.0 ± 72.1 f	3451.7 ± 92.8 e
	N75	CN	2.6 ± 0.0 bc	43.1 ± 1.8 ab	3253.5 ± 14.9 e	4436.0 ± 47.3 d
		PN	2.6 ± 0.0 bc	47.3 ± 3.7 a	3924.5 ± 69.3 d	4900.0 ± 42.4 bc
	N112.5	CN	2.7 ± 0.0 abc	43.3 ± 2.4 ab	3228.6 ± 44.0 e	4582.7 ± 157.5 cd
		PN	2.7 ± 0.1 abc	46.7 ± 3.1 a	3836.6 ± 46.3 d	5323.7 ± 45.7 b
	N150	CN	2.7 ± 0.0 abc	41.5 ± 2.0 abc	3701.8 ± 56.0 d	5204.2 ± 17.0 b
		PN	2.7 ± 0.0 ab	44.3 ± 0.5 ab	4406.0 ± 109.5 c	5878.5 ± 78.5 a
	N225	CN	2.7 ± 0.0 abc	37.5 ± 2.0 bcde	3848.1 ± 126.6 d	4728.6 ± 80.5 cd
		PN	2.7 ± 0.0 abc	40.7 ± 1.2 abcd	4575.3 ± 86.4 c	5221.8 ± 154.7 b
	N337.5	CN	2.6 ± 0.0 c	32.4 ± 2.7 de	5946.0 ± 128.8 a	4590.1 ± 171.4 d
		PN	2.7 ± 0.0 abc	33.3 ± 4.9 cde	5452.3 ± 240.2 b	5244.5 ± 216.3 b
ANOVA						
Year			**	**	*	**
N level			ns	**	**	**
Treatment			ns	ns	**	**

Note: CN: conventional nitrogen fertilizer treatment; PN: polyaspartic acid–chitosan with nitrogen fertilizer treatment. N0, N75, N112.5, N150, N225, and N337.5 represent nitrogen application amount of 0, 75, 112.5, 150, 225, and 337.5 kg N ha^−1^, respectively. Data represent mean ± SE. Different letters within a column mean significant difference among treatments at *p* < 0.05 according to DANCUN. * and ** represent significant difference at *p* < 0.05 and *p* < 0.01, respectively, and ns represents no significant difference.

**Table 2:** Foxtail millet N partial factor productivity efficiency (NPEP), N agronomic efficiency (NAE), N recovery efficiency (NREN), and N physiological efficiency (NPE) at five N rates under the control (CN) and polyaspartic acid–coated urea (PN) in 2020 and 2021.

Year	Nitrogen Application Level	Treatment	NPFP (kg kg^−1^)	NAE (kg kg^−1^)	NRE (%)	NPE (kg kg^−1^)
2020	N75	CN	64.3 ± 2.2 b	26.4 ± 2.2 b	64.0 ± 1.2 cd	41.1 ± 0.9 a
		PN	68.0 ± 0.6 a	32.5 ± 0.6 a	78.7 ± 1.0 a	41.2 ± 0.2 a
	N112.5	CN	41.8 ± 0.4 d	16.6 ± 0.4 d	68.6 ± 1.7 b	24.2 ± 0.3 d
		PN	48.4 ± 1.6 c	24.8 ± 1.6 b	79.9 ± 0.7 a	31.0 ± 0.8 b
	N150	CN	34.6 ± 2.1 e	15.7 ± 2.1 d	68.2 ± 0.9 bc	22.9 ± 0.3 d
		PN	38.7 ± 1.3 d	21.0 ± 1.3 c	79.5 ± 2.5 a	26.3 ± 0.8 c
	N225	CN	23.7 ± 0.1 f	11.0 ± 0.1 ef	61.9 ± 0.4 d	17.8 ± 0.0 f
		PN	24.8 ± 0.5 f	12.9 ± 0.5 de	66.4 ± 1.2 bc	19.4 ± 0.5 e
	N337.5	CN	15.9 ± 0.3 g	7.5 ± 0.3 f	46.5 ± 0.8 e	16.0 ± 0.3 g
		PN	16.8 ± 0.7 g	8.9 ± 0.7 f	44.4 ± 1.9 e	19.9 ± 0.7 e
2021	N75	CN	59.2 ± 0.6 b	13.9 ± 0.6 c	54.4 ± 3.8 e	25.6 ± 1.5 b
		PN	65.3 ± 0.6 a	19.3 ± 0.6 a	70.3 ± 6.6 bc	27.7 ± 2.3 a
	N112.5	CN	40.7 ± 0.3 d	10.6 ± 0.3 e	56.2 ± 11.2 e	19.3 ± 2.4 d
		PN	47.3 ± 0.4 c	16.6 ± 0.4 b	78.7 ± 0.5 ab	21.1 ± 0.6 c
	N150	CN	34.7 ± 0.1 f	12.1 ± 0.1 d	66.4 ± 2.8 cd	18.2 ± 0.3 d
		PN	39.2 ± 0.5 e	16.2 ± 0.5 b	84.9 ± 3.3 a	19.1 ± 1.0 d
	N225	CN	21.0 ± 0.4 h	5.9 ± 0.4 g	63.7 ± 8.1 cde	9.4 ± 0.5 e
		PN	23.2 ± 0.7 g	7.9 ± 0.7 f	80.0 ± 2.2 ab	9.8 ± 0.8 e
	N337.5	CN	13.6 ± 0.5 j	3.5 ± 0.5 h	59.9 ± 2.2 de	5.9 ± 0.8 f
		PN	15.5 ± 0.6 i	5.3 ± 0.6 g	76.5 ± 1.6 ab	7.0 ± 0.9 f
ANOVA						
Year			ns	**	ns	**
N level			**	**	**	**
Treatment			*	*	ns	*

Note: CN: conventional nitrogen fertilizer treatment; PN: polyaspartic acid–chitosan with nitrogen fertilizer treatment. N0, N75, N112.5, N150, N225, and N337.5 represent nitrogen application amount of 0, 75, 112.5, 150, 225, and 337.5 kg N ha^−1^, respectively. Data represent mean ± SE. Different small letters within a column mean significant difference among treatments at *p* < 0.05 according to DANCUN. * and ** represent significant difference at *p* < 0.05 and *p* < 0.01, respectively, and ns represents no significant difference.

## Data Availability

Data are available from the corresponding author.
